# Intraluminal MRI and interventions: Innovation and application

**DOI:** 10.1016/j.engmed.2024.100044

**Published:** 2024-11-25

**Authors:** Feng Zhang, Grace Laidlaw, Guy Johnson, Hugh McGregor, Hongxiu Ji, Xiaoming Yang

**Affiliations:** Image-Guided Bio-Molecular Intervention Research and Division of Interventional Radiology, Department of Radiology, University of Washington School of Medicine, Seattle, USA

**Keywords:** Radiology, Magnetic resonance imaging, Intraluminal, Interventional, Image-guided

## Abstract

Intraluminal magnetic resonance imaging (MRI) is a promising option to guide interventions, offering several advantages over other imaging modalities. It provides high spatial and contrast resolution for imaging luminal structures, excellent extra-luminal soft tissue visualization, real-time tracking of interventional devices, and operates without ionizing radiation. The applications of intraluminal MRI range from high-resolution imaging of vessel walls to MRI-guided interventions for managing life-threatening conditions such as cardiovascular atherosclerotic disease and malignancies within luminal structures. Clinical use of intraluminal MR technology optimizes endovascular delivery of therapeutics to targeted vessel segments and guides myocardial delivery of stem cells. However, advancements are still required, such as the refinement of MR-compatible interventional devices, development of real-time “MR fluoroscopy” similar to X-ray fluoroscopy, and establishment of safe clinical environments with large bore and short magnets. These improvements are essential for broader clinical adoption of intraluminal MR technology in healthcare.

## Introduction

The goal of intraluminal imaging is to visualize the inner surface and wall of luminal structures. This began with the generation of inside-out views of vasculature through minimally invasive imaging techniques, including angioscopy [1,2], intravascular ultrasound (IVUS) [3,4], and optical coherence tomography [5,6]. Recently, non-invasive methods like computed tomography (CT) [7], magnetic resonance imaging (MRI) [8], and positron emission tomography (PET) [9], have been used. CT detects high-density biological tissues (such as vascular wall calcification), while MRI excels in characterizing vessel wall architecture in accessible areas like the carotid arteries, using either external or surface coils. 2′-Deoxy-2′-(18F)-fluoro-d-glucose (18F-FDG) PET/CT can detect inflammation in atherosclerosis: arterial wall 18F-FDG uptake is associated with macrophage content and inflammatory gene expression in plaques. 18F-FDG-PET/CT uptake in plaques strongly predicts future cardiovascular events in patients with atherosclerosis [10]. However, PET is limited in guiding treatment procedures for vascular diseases.

**Fig. 1.**
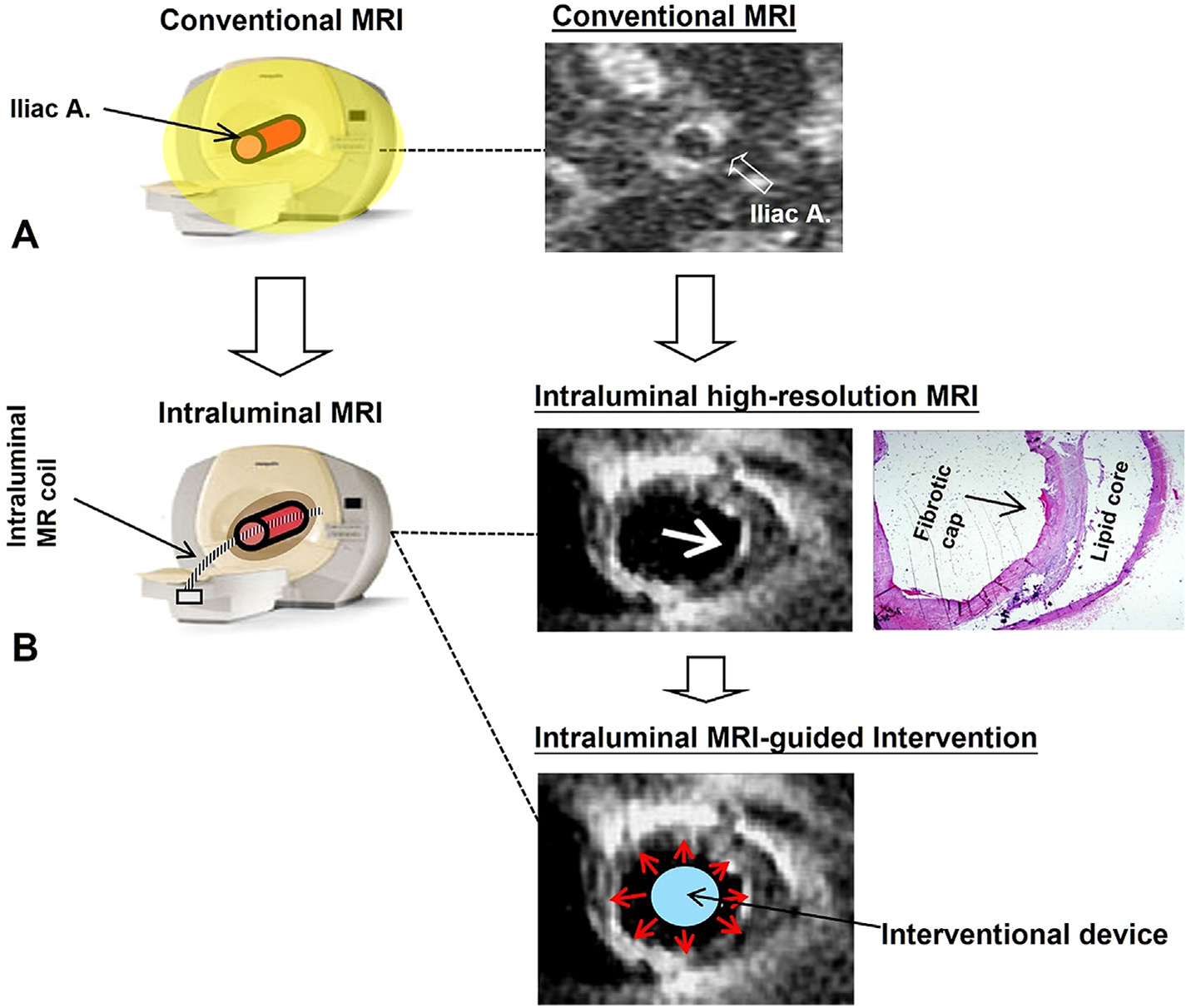
A comparison of conventional MRI and intraluminal MRI. (A) Conventional MRI of an iliac artery. The broad MR signal (yellow) acquired during conventional MRI creates a cross-sectional image of the artery but does not resolve the apparent discontinuity in the hyperintense arterial wall at 3 o'clock (open arrow). (B) Intraluminal MRI. An intraluminal receiver coil positioned in the same artery captures a highly localized MR signal. Intraluminal MRI shows that the arterial wall discontinuity is due to an atherosclerotic plaque with a fibrotic cap and lipid core, confirmed by pathology. The intraluminal MR coil requires interventional devices, offering opportunities for intraluminal MR-guided interventions, potentially delivering therapeutic interventions (red arrows) into the target atherosclerotic plaque to prevent progression.

Atherosclerotic plaque composition is a key factor in assessing vulnerability to rupture. While current imaging techniques can evaluate superficial atherosclerotic lesions, they lack sensitivity for detecting plaques in deeper vessels commonly affected by atherosclerosis, like the iliac, renal, and coronary arteries. Traditionally, accurate biological characterization of atherosclerotic lesions in these vessels required biopsy or autopsy. Intraluminal MRI overcomes these limitations by using a “local” MR receiver coil placed inside the vessel near an atherosclerotic lesion, known as “inside-out MRI,” which enhances signal capture and allows for high-resolution imaging of the vessel wall. This innovative concept was initially described by Kantor et al. for detailing the myocardium [11,12]. Intraluminal MRI has also been applied to non-vascular luminal structures, such as bile ducts and the urethra. The use of therapeutic devices under intraluminal MRI guidance has led to the concept of “intraluminal MRI-guided interventions” (Fig. 1).

## Intraluminal MRI devices

Intraluminal MR devices are classified as “passive” or “active.” Passive MR devices are visible on MRI, while active devices enable high-resolution image acquisition [13].

### Passive MR devices

Passive MR devices, which are not directly integrated into MRI systems, are visible due to their intrinsic material properties, creating either negative (dark) or positive (bright) signal contrast through magnetic field distortion [14]. These devices can be categorized by contrast mechanisms based on local field inhomogeneity or susceptibility effects [12]. Devices based on field inhomogeneity use a solenoid coil mounted on the tip of an interventional instrument (e.g., guidewire or catheter) or a copper wire mounted along the instrumenťs length [15]. A solenoidal coil or copper wire generates a magnetic field when current passes through, making it visible on MRI [16,17].

Susceptibility-based devices, incorporating ferromagnetic/paramagnetic materials such as dysprosium oxide, glass fiber rings, or other MRI-visible markers (air, gadolinium, or carbon dioxide) onto interventional instruments, cause local signal loss, allowing the instrumenťs impregnated parts to be visualized by MRI [18–20].

Passive MR devices are valued for simplicity and safety, with no risk of radiofrequency (RF)-induced heating; however, they may suffer from low contrast-to-noise ratios, limiting anatomical distinction [14].

### Active MR devices

**Fig. 2.**
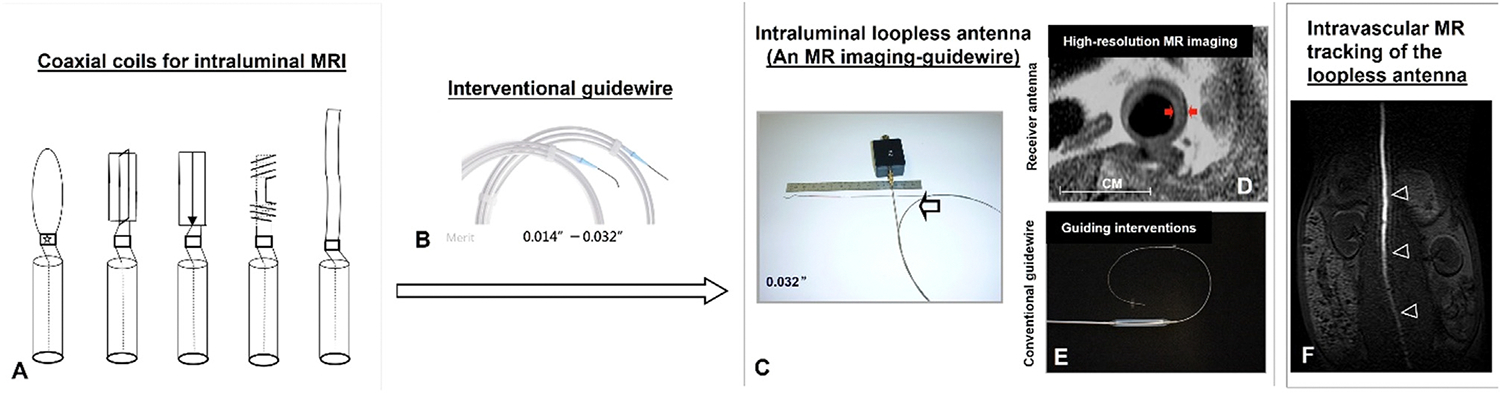
(A) Early active MR receivers with coaxial cables and looped coil ends of various geometries for intravascular MRI. (B) A loopless antenna evolved from looped MR coils, mimicking guidewires and produced at standard sizes (0.014–0.032 inches in diameter) (C). (D&E) The loopless antenna functions as an MR imaging guidewire, facilitating intravascular high-resolution MRI of the thickened vessel wall (between arrows in D) and guiding endovascular interventions like balloon angioplasty (E). (F) The full length of the MR imaging guidewire (arrowheads) can be tracked in the aorta under MRI.

Active MR devices, equipped with embedded RF coils, antennas, or other sensors, function as internal receivers. Early active MR devices for intravascular MRI used coaxial cables with radiofrequency coil tips in various geometries (Fig. 2) [12]. Proximity of the MR detector coil to the arterial wall in intravascular MRI (IVMRI) enhances image quality, allowing detailed structure characterization of deep arteries. Studies have shown that active MR devices for IVMRI improve signal-to-noise ratio (SNR) by 10–20 fold compared to body or external MR coils [21].

However, early active endoluminal receivers were rigid and too large for small, tortuous vessels like coronary arteries. Modern receivers have been refined by adopting a “loopless” configuration, making them suitable for smaller structures [22]. The involvement of interventional radiologists in designing MR-compatible instruments has led to loopless antennas resembling conventional guidewires with 0.014–0.035-inch diameters (Fig. 2).

These loopless antennas can be combined with commercially available interventional devices, such as needles and catheters, for interventional procedures like local therapeutic infusion, balloon angioplasty, and stent placement (Fig. 2). Conversion of active MR receivers from a looped coil to a flexible loopless antenna has created “MR imaging guidewires” (MRIGs), capable of generating intravascular high-resolution MRI and guiding interventions [23]. MRIGs, as small as 0.014 inches in diameter, facilitate small-vessel interventions, such as intracoronary MRI-guided procedures [24,25]. Additional advantages include full-length wire visualization under MRI, essential for safe interventional procedures [26].

## Applications

To date, intraluminal MRI has been used in both pre-clinical and clinical settings. Initially applied to cardiovascular diseases (such as atherosclerosis and cardiac arrhythmias), new applications are emerging in non-cardiovascular diseases (e.g., biliary and urinary malignancies).

### Intravascular MRI

**Fig. 3.**
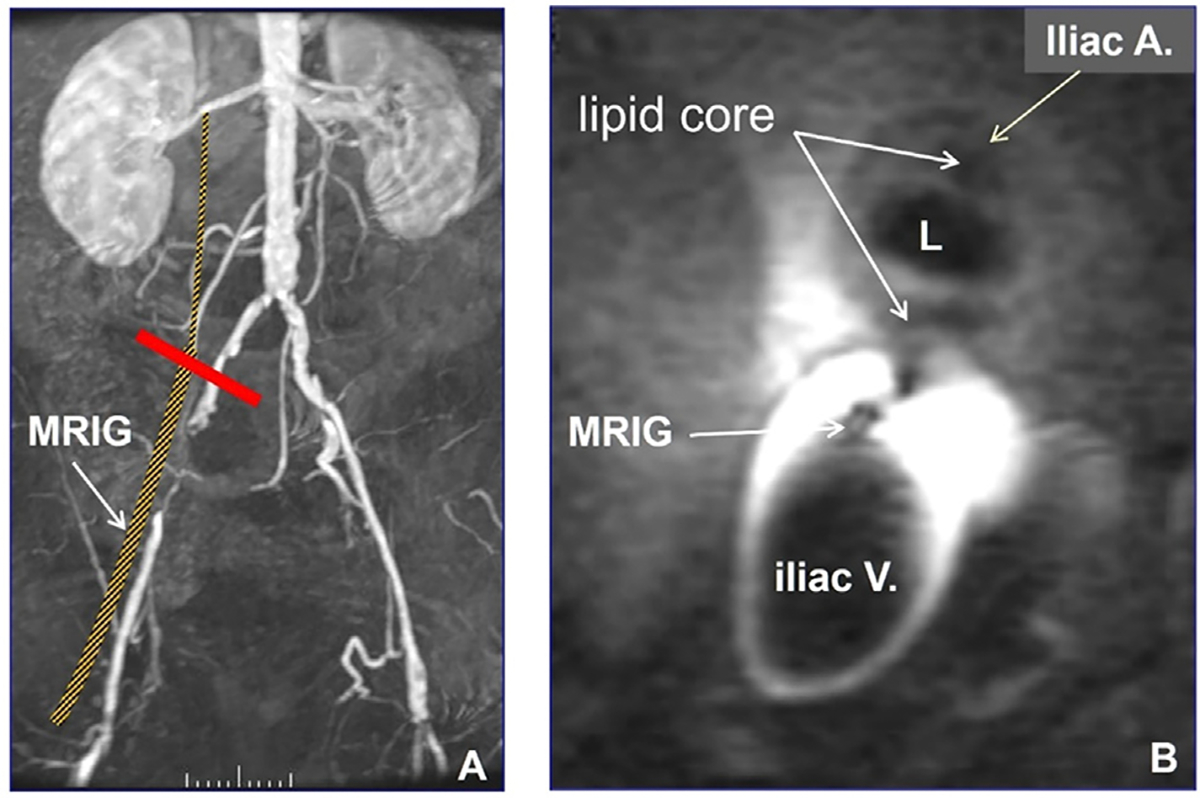
Transvenous high-resolution MRI of atherosclerotic plaque in an iliac artery. (A) The MR imaging-guidewire (MRIG) is positioned in the iliac vein to obtain a cross-sectional view (red line) of the adjacent iliac artery. (B) The transvenous cross-sectional MRI captures high MR signal with the MRIG and depicts an atherosclerotic plaque with a lipid core narrowing the iliac artery lumen (L) (Courtesy of Lawrence Hofmann).

The first study using intravascular MRI with an active MR imaging guidewire (MRIG), or loopless antenna, involved positioning the MRIG in the iliac vein, which allowed for high-resolution imaging of the adjacent iliac artery wall [27]. This transvenous approach provides the imaging equivalent of *in-vivo* pathology, characterizing atherosclerotic features, such as plaque with a fibrous cap and lipid core (Fig. 3).

Another similar technique, transesophageal MRI of the aorta, also functions as intraluminal MRI with MRIG positioning in the esophagus achieving high-resolution imaging of atherosclerotic plaques in the adjacent aortic arch and descending thoracic aorta, both anatomically close to the esophagus [28].

For diagnostic imaging, a primary reason to avoid direct MRIG placement in the target vessels is to reduce the risk of disturbing atherosclerotic plaque during MRIG manipulation, which could lead to distal arterial embolization of plaque fragments, especially in diseased arteries with vulnerable lesions.

### Intravascular MRI-guided interventions

Loopless MR antennae can serve as both guidewires and MRI signal receivers, and they have been used in guiding intravascular interventions such as balloon angioplasty [29,30], stent placement [19], and gene therapy [31,32].

Catheter-based therapeutic delivery offers a promising method to localize high doses at targeted sites while minimizing systemic toxicity. For example, controlled heating has been shown to improve the effectiveness of locally delivered therapeutics [33]. However, whole-body heating is impractical in clinical settings. A viable alternative is to use a small internal heating source, which can be navigated to a target site through natural anatomic channels like blood vessels [34,35]. Thus, MRIGs have been further developed into “MR imaging--heating-guidewires” (MRIHGs), where the loopless MR antenna also serves as an intravascular RF heating source, creating localized heating at the target site to enhance therapeutic delivery effectiveness.

**Fig. 4.**
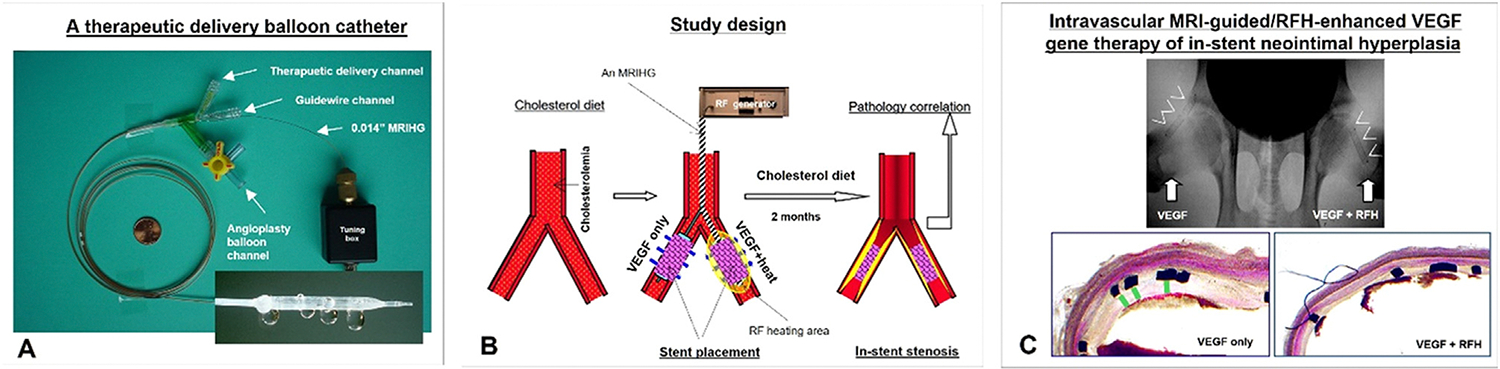
Intravascular MRI-guided and RF heat-enhanced VEGF gene therapy for in-stent restenosis. (A) A multifunctional balloon catheter with [1] a channel for balloon inflation against the vessel wall [2], a channel for local infusion of therapeutics through the balloon's micro-pores under MRI guidance, and [3] a guidewire channel for positioning the MR imaging-heating-guidewire (MRIHG). (B) Protocol for MRI-guided and RFH-enhanced VEGF therapy to inhibit in-stent neointimal hyperplasia, including 1) a high-cholesterol diet, 2) VEGF delivery to bilateral femoral-iliac arteries with MRIHG-mediated RFH on the left, 3) stent placement in targeted segments, 4) continuation of diet, and 5) tissue harvesting for pathology. (C) Comparison between VEGF-only treated right femoral artery and VEGF/RFH-treated left femoral artery. (Upper panel) Radiographs obtained two months post-stenting; (Lower panel) histology shows neointimal hyperplasia (green lines) in the VEGF-only treated artery, absent in the VEGF/RFH-treated artery.

One example of MRIHG application is in the endovascular delivery of vascular endothelial growth factor (VEGF), aimed at increasing local VEGF activity to prevent in-stent restenosis (Fig. 4) [36].

### Intrabiliary MRI

**Fig. 5.**
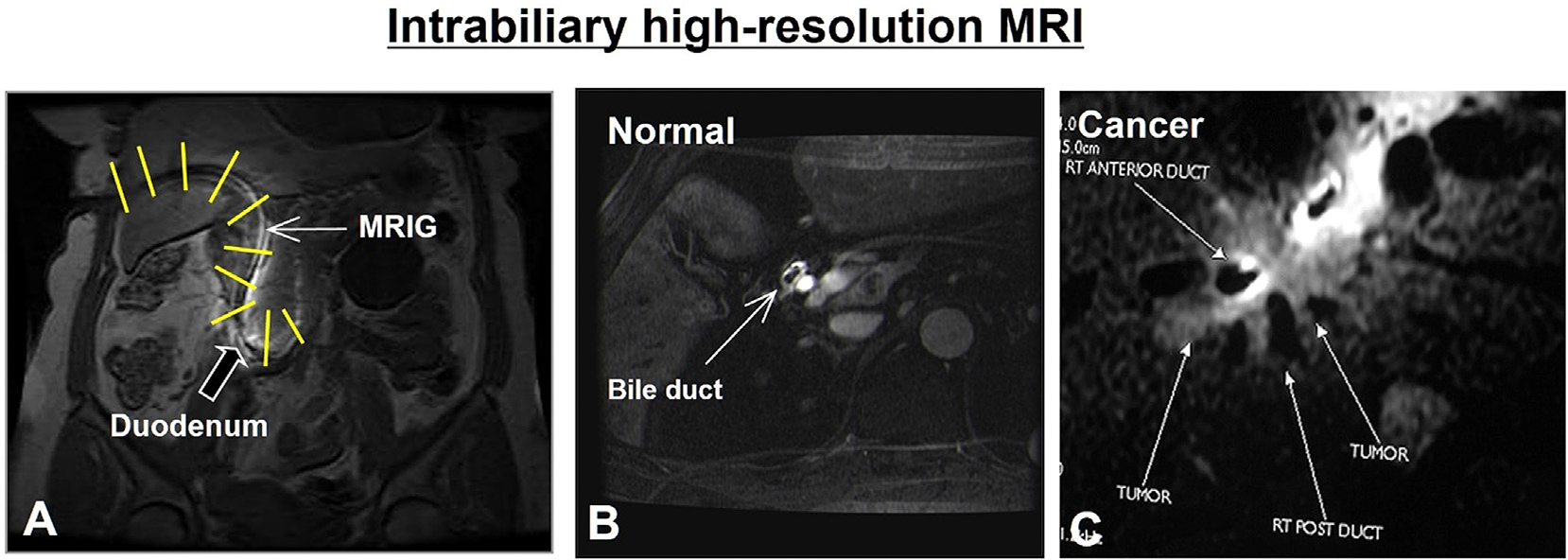
Intrabiliary MRI. (A) Coronal MRI showing MRIG (arrow) entry into the right bile duct, common bile duct, and duodenum through a percutaneous biliary drain, with intrabiliary MRIs captured along the MRIG (yellow lines). (B) Intrabiliary cross-sectional MRI of a normal bile duct as a “clean bright ring” (arrow B), compared to a bile duct with tumor infiltration (C) (Courtesy of Aravind Arepally).

The promising results of intravascular MRI and related interventions have encouraged exploring intraluminal MRI in non-vascular structures, such as the biliary system. The small size, deep location, and complex anatomy of the common bile duct (CBD) make in vivo assessment of its walls challenging, and therapeutic delivery to the CBD walls has also been difficult. To address this, intraluminal MRI was first applied to the biliary system by placing an MRIG in the bile duct via a percutaneous biliary drain [37]. Compared to conventional MRI, intrabiliary MRI, performed via percutaneous biliary access, allows for a smaller field of view with higher in-plane resolution and excellent contrast between the biliary lumen and surrounding structures, improving diagnostic accuracy for challenging biliary malignancies like cholangiocarcinoma (Fig. 5) [38]. Intrabiliary MRI also helps visualize the CBD wall and accurately detect tumor extension [39].

### Intrabiliary MRI-guided interventions

**Fig. 6.**
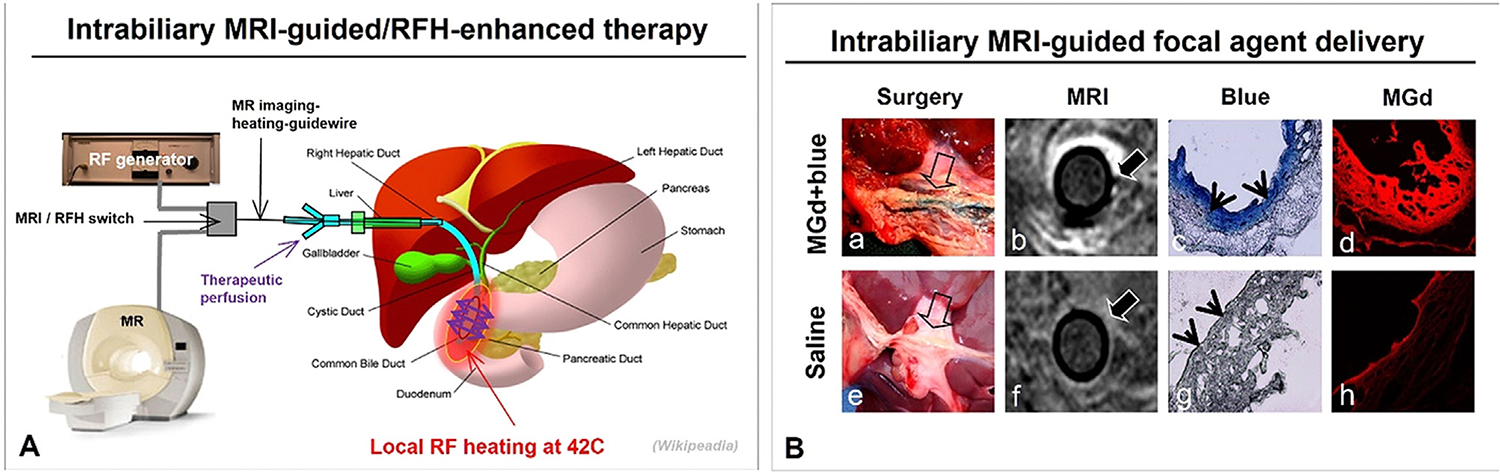
(A) Intrabiliary MRI-guided and RF heat-enhanced direct therapy of biliary malignancies. The MR imaging-heating-guidewire allows high-resolution intrabiliary MRI, direct therapeutic delivery to the biliary wall, and localized RF heating for enhanced efficacy. (B) Intrabiliary delivery of (a–d) a motexafin gadolinium (MGd)/trypan blue mixture versus saline (e–h). Surgical images reveal (a) blue-stained CBD with MGd infusion (arrow), absent in (e) control CBD with saline. MR images show (b) high signal intensity in the CBD wall and peri-biliary tissue with MGd, absent with (f) saline. Histology confirms blue dye (arrowheads on c) and MGd as red fluorescence (d) in the CBD wall, absent in the control CBD (g, h). Autofluorescence is visible in (h).

Primary biliary malignancies are often challenging to treat surgically and carry significant morbidity and mortality risks, even in resectable cases. Thus, combining intrabiliary MRI with interventional oncology techniques is under investigation. The use of intrabiliary MRIHGs enables simultaneous imaging guidance and thermal enhancement during direct anti-tumor agent delivery to the biliary wall (Fig. 6) [40–42]. MRIHGs can deliver thermal energy to improve perfusion and therapeutic penetration into bile duct walls. This intrabiliary RFH-enhanced therapy technique may hold significant potential for efficiently treating biliary malignancies [41].

### Intraurethral MRI

**Fig. 7.**
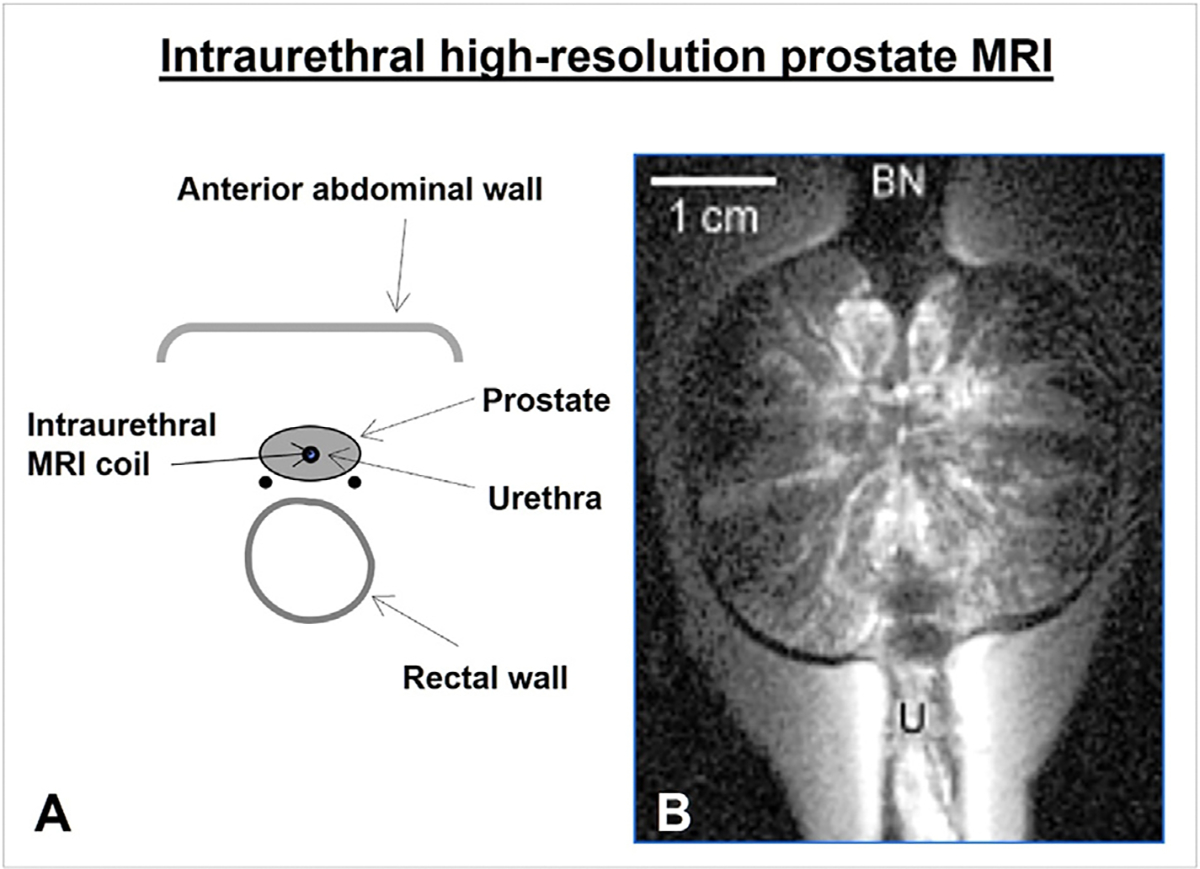
Intraurethral prostate MRI. (A) A graphic of the pelvis shows the intraluminal MRI coil positioned within the urethra to achieve transurethral high-resolution MRI of the adjacent prostate (B). BN = bladder neck; U = urethra. (Courtesy of Ergin Atalar).

Intraluminal MR technology has also been applied to the genitourinary system. For instance, an intraluminal MR coil or MRIG can be placed in the urethra to generate high-resolution intraurethral MRI images of the prostate or periurethral tissues (Fig. 7) [43–45].

### Intraluminal MRI-guided genitourinary interventions

**Fig. 8.**
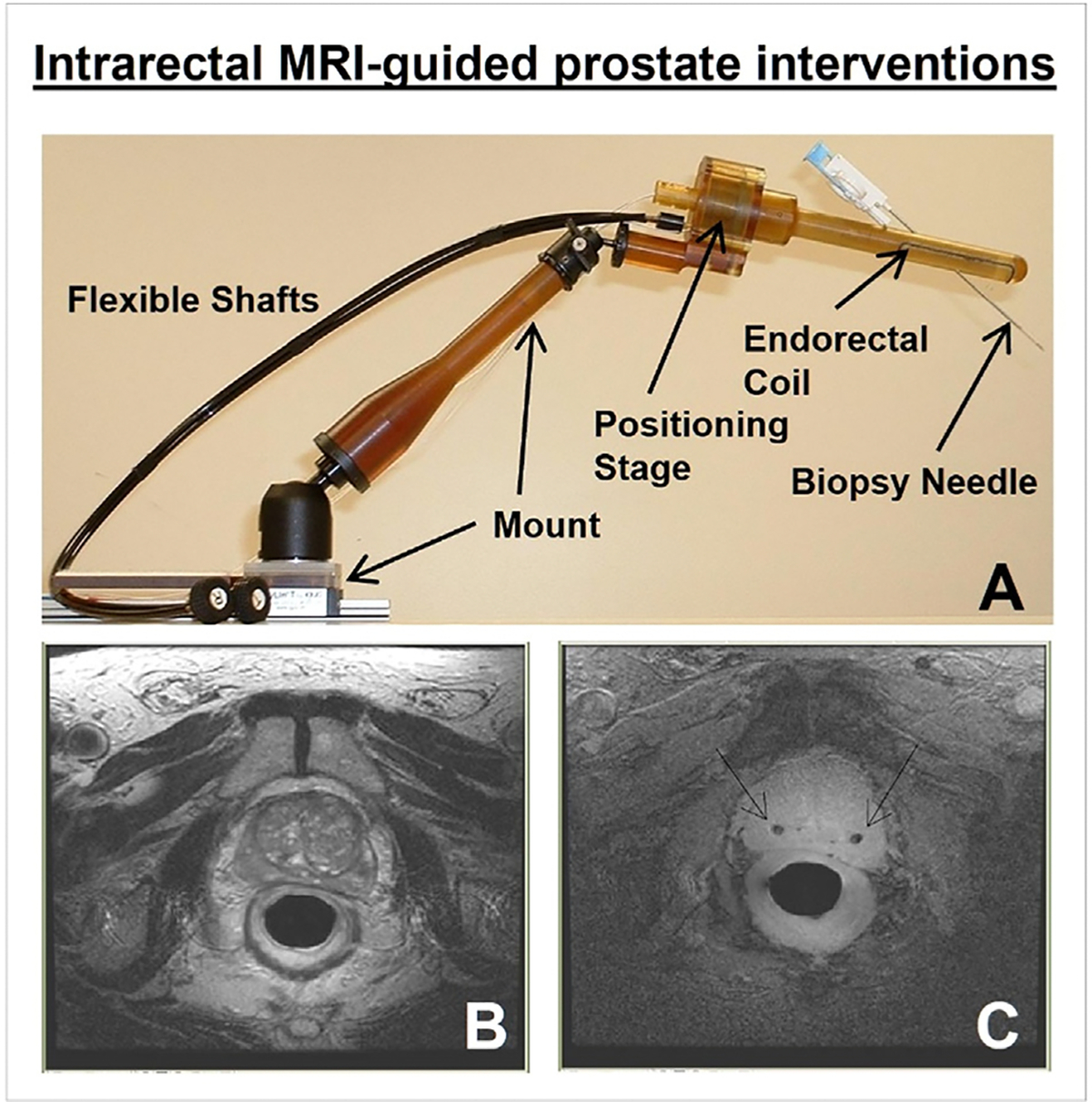
(A) A transrectal MRI device with a needle guide and sheath, positioning stage, insertion stage, flexible actuation shafts, and mount. (B) Transrectal cross-sectional high-resolution MRI of the prostate, using the manipulator for precise insertion of two radiation seeds (arrows in C) into the prostate for internal radiation therapy (Courtesy of Robert Susil).

Intraluminal MRI-guided interventions in the genitourinary system are typically performed by placing an endorectal coil or MRIG to guide prostate interventions, including biopsy, brachytherapy seed placement, and positioning of interventional oncologic devices for tissue ablation [46–49]. One example is the “Artemis fusion device,” which stabilizes the positioning of laser fibers and thermal probes under endorectal MRI guidance [50]. More recent advancements involve robot-assisted prostate interventions within the MRI bore. This technique enables MRI-guided intervention in a closed-loop system, with real-time tracking of tissue deformation, target movement, instrument localization, and therapy delivery (Fig. 8) [51].

### Intracardiac MRI-guided interventions

MRI-guided interventions have enhanced the management of various cardiovascular diseases, including cardiac arrhythmias [54–58], congenital and structural heart disease [52,53], coronary artery disease [54,55], and pulmonary hypertension [56]. Recent developments also include the use of artificial intelligence (AI) in MRI-guided cardiac catheterization [57,58].

The advantages of real-time MRI guidance over conventional electrophysiology in treating cardiac arrhythmias include simultaneous three-dimensional substrate assessment, along with accurate intra-procedural guidance and evaluation of target lesions. These benefits support the growing field of MRI-guided electrophysiology, facilitating effective intracardiac ablation of ectopic tissue causing arrhythmias and allowing real-time visualization of ablation-related tissue interactions [59]. Passive and active devices have been developed for intracardiac MRI-guided ablation and are now used clinically [60–62].

**Fig. 9.**
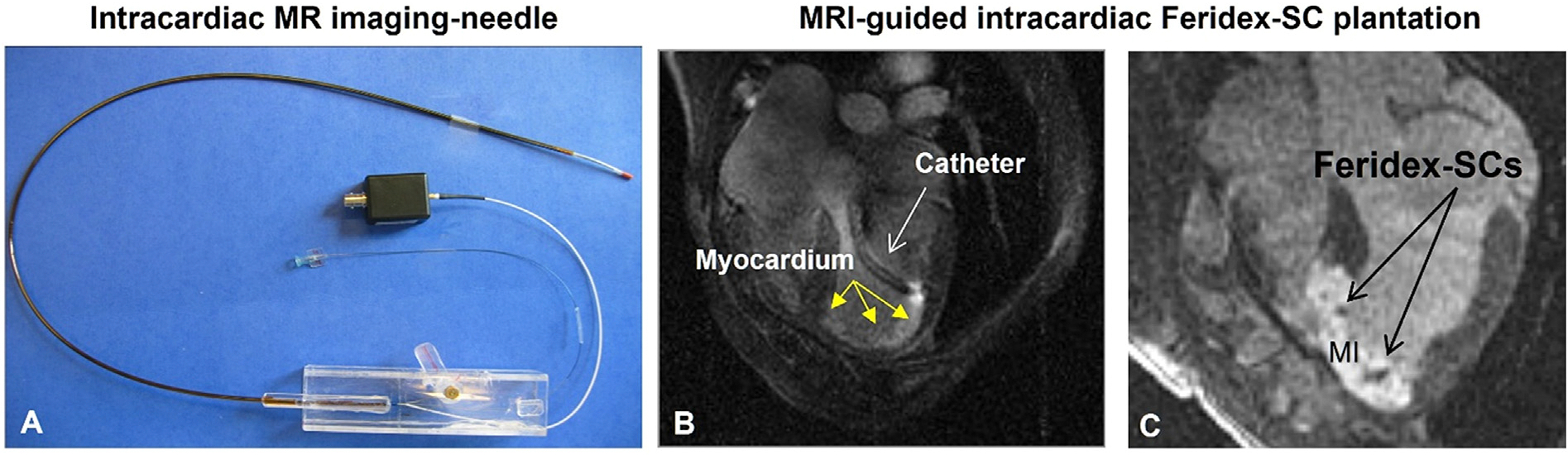
MRI-guided intracardiac stem cell (SC) injection for myocardial ischemia (MI) treatment. (A&B) An active MRI-compatible catheter for intracardiac injection of therapeutic agents or stem cells into the myocardium under MR guidance (Courtesy of Parag Karmarkar). (C) Long-axis MRI showing a hypointense SC cluster (arrows), resulting from SCs labeled by a T2-MR contrast agent, Feridex (Courtesy of Ergin Atalar).

Another promising advancement in MRI-guided cardiac interventions is the intracardiac delivery of stem cells for regenerative repair of ischemic heart disease [63]. The development of an MR-trackable intramyocardial injection catheter has enabled direct injection of therapeutic agents, including stem cells, into the myocardium (Fig. 9) [64,65].

A recent advancement is the development of a novel passive MR-compatible bioptome for MR-guided intracardiac biopsy [65]. The orientation of MR-compatible bioptomes relative to the myocardium is clearly visualized under MRI, enabling more precise targeting of specific myocardial areas compared to conventional X-ray fluoroscopy.

## Summary

Intraluminal MRI is a promising technique for guiding interventions, offering several advantages over other imaging modalities, including high spatial resolution and tissue contrast for luminal wall imaging, excellent three-dimensional soft tissue visualization, real-time target tracking, and an absence of radiation risk. The development of intraluminal MR technology, as described in this review, demonstrates successful multidisciplinary collaboration in medical and engineering fields. This technology relies on the intraluminal placement of MR receivers and has applications in managing cardiovascular diseases and non-vascular conditions affecting luminal structures, such as those in the biliary and genitourinary systems. Intraluminal MR technology has already translated into clinical practice, advancing modern medicine, including gene and stem cell therapies. Ongoing technical improvements, such as refining MR-compatible interventional devices, developing real-time MR fluoroscopy to match X-ray fluoroscopy, and creating a safe clinical environment with large bore and short magnet scanners, should further facilitate the broad clinical application of intraluminal MR technology.
